# Hyperthermic Intrathoracic Chemoperfusion and the Role of Adjunct Immunotherapy for the Treatment of Pleural Mesothelioma

**DOI:** 10.3390/biom15050678

**Published:** 2025-05-07

**Authors:** Susan Luozheng Kong, Zihan Feng, Sangmin Kim, Edra K. Ha, Kero Kamel, Michael Becich, James D. Luketich, Arjun Pennathur

**Affiliations:** 1 Department of Cardiothoracic Surgery, University of Pittsburgh School of Medicine, Pittsburgh, PA 15213, USA; kongl2@upmc.edu (S.L.K.); kamelk@upmc.edu (K.K.); luketichjd@upmc.edu (J.D.L.); 2 Department of Surgery, University of Pittsburgh School of Medicine, Pittsburgh, PA 15213, USA; fengz2@upmc.edu; 3 Department of Bioinformatics, University of Pittsburgh School of Medicine, Pittsburgh, PA 15213, USA; becichmj@upmc.edu; 4 UPMC Hillman Cancer Center Pittsburgh, Pittsburgh, PA 15213, USA

**Keywords:** pleural mesothelioma, hyperthermic intrathoracic chemoperfusion, immunotherapy

## Abstract

Pleural mesothelioma (PM) is an aggressive cancer originating from the mesothelial lining of the pleura, with a rising global incidence since the mid-20th century due to asbestos and erionite exposure. PM accounts for 80–90% of all mesothelioma cases and is histologically classified into three subtypes—epithelioid, sarcomatoid, and biphasic— with epithelioid carrying the most favorable prognosis. Despite advances in surgery, chemotherapy, radiotherapy, and immunotherapy, PM prognosis remains poor, necessitating more effective, multimodal strategies. Hyperthermic intrathoracic chemoperfusion (HITHOC) has emerged as a promising adjunct to cytoreductive surgery by delivering heated chemotherapy directly to the pleural cavity, potentially improving survival—especially in patients with epithelioid PM. Combining HITHOC with post-surgical immunotherapy represents a novel approach to enhancing both local and systemic anti-tumor responses and targeting microscopic disease and distant metastases. This review explores surgical outcomes after surgery for PM, the therapeutic synergy of HITHOC and immunotherapy, ongoing clinical trials evaluating this multimodal strategy, and its implications for future patient care.

## 1. Introduction and Background

Malignant pleural mesothelioma (PM) is a rare and aggressive cancer arising from the mesothelial lining of the pleura, with 60% of patients presenting with symptoms of pleural effusion, dyspnea, and chest wall pain [1]. Its incidence has been rising worldwide since the mid-20th century, closely linked to breathing in asbestos and erionite fibers [2]. Asbestos was widely used in industry in most developed countries and is still present in many older buildings to this day [3]. Asbestos exposure remains the primary risk factor for PM, with a clear dose-dependent relationship [3]. Due to the remarkably long latency period, which could last for 30–40 years between the first exposure to asbestos and the onset of symptoms, mesothelioma has not received sufficient attention, and the lack of more effective treatments and medications in the past has led to its survival rate remaining largely unaltered for the past 50 years [4].

PM accounts for approximately 80–90% of all mesothelioma cases [2]. The global age-adjusted mortality rate for mesothelioma is 4.9 per million patients, with a mean age at death of 70 years old and a male-to-female ratio of 3.6 to 1 [2,5]. Grossly, mesothelioma may first appear on the parietal pleura as multiple small grape-like nodules. Macroscopically, focal necrosis or hemorrhage may be seen. Histologically, PM is classified into three main subtypes—epithelioid (the most common, accounting for 60% of cases), sarcomatoid (10–20% of cases), and biphasic (20–30% of cases) [3]. The epithelioid subtype generally has a better prognosis as compared with the other subtypes [3], but it is unknown what leads to the different subtypes, and analysis of more than 2000 mesothelioma cases failed to identify any association with different asbestos fiber types, latency or duration, or cumulative exposure characteristics [6].

Current treatments for PM include a multimodal approach including surgery, chemotherapy, radiotherapy, and immunotherapy, according to the European Society for Medical Oncology (ESMO) Clinical Practice Guidelines and National Comprehensive Cancer Network (NCCN) Guidelines [7,8]. Despite these options, prognosis remains poor, driving the search for more effective combinations of multimodal approaches. Hyperthermic intrathoracic chemoperfusion (HITHOC) has emerged as a promising adjunct to cytoreductive surgery because it delivers heated chemotherapy directly to the pleural cavity. This technique aims to enhance local drug concentrations and cytotoxicity while minimizing systemic side effects [9]. Recent studies suggest that HITHOC may be associated with improved survival outcomes, particularly for patients with epithelioid PM [10]. Combining HITHOC with immunotherapy after surgery represents a novel strategy to leverage both local and systemic anti-tumor responses, potentially addressing microscopic disease and distant metastases [11]. There is still a significant need for further research to optimize protocols and explore the potential synergies with immunotherapy [12]. In this review, we summarize the results of surgery for PM, and explore the potentially synergistic therapeutic effects of HITHOC combined with immunotherapy after surgery, current ongoing clinical trials that test the efficacy of this multimodal approach, and their implications for future PM patient care.

## 2. State-of-the-Art Treatment Paradigm for PM

All patients diagnosed with mesothelioma should be evaluated for multimodality treatments including surgery often combined with chemotherapy and/or radiotherapy for resectable tumors, and systemic therapy alone for unresectable tumors [13]. Treatment decisions should ideally be made within a multidisciplinary tumor board, and factors including staging, histology, comorbidities, age, and patient preferences should be considered [14].

### 2.1. Surgical Treatment of PM

Several society guidelines recommend surgery as a component of diagnosis and/or treatment of PM [15]. Selected patients diagnosed with mesothelioma are candidates for curative surgery. Factors taken into consideration for selection for curative surgery includes the stage and the extent of the disease and the presence of comorbidities. Surgery aims to achieve a maximal cytoreduction or a macroscopic complete resection (MCR). The main surgical options are either extrapleural pneumonectomy (EPP) or pleurectomy/decortication (PD), which involves total pleurectomy and decortication with or without resection of the pericardium and diaphragm. Extended pleurectomy/decortication (EPD) refers to resection of the diaphragm and pericardium in addition to total pleurectomy [16]. Mediastinal lymph node dissection is recommended with EPP or pleurectomy/decortication. While EPP involves *en bloc* resection of the lung, pleura, pericardium, and diaphragm, EPD is a lung-sparing procedure that removes only the pericardium, hemidiaphragm, and parietal and visceral pleurae. In a systematic review of 1145 patients, Cao et al. found that selected patients who underwent EPD had lower perioperative morbidity (27.9% vs. 62.0%, *p* < 0.001) and mortality (2.9% vs. 6.8%, *p* = 0.02) with similar overall survival compared with those who underwent EPP [17]. Similarly, Taioli et al. reported in their meta-analysis that EPD is associated with a 2.5-fold lower short-term mortality (perioperatively and within 30 days) than EPP, and the overall survival was similar 2 years after surgery [18]. Luckraz et al. also concluded in their 30-year series that EPD combined with adjuvant therapy provided better overall survival compared with EPP [19]. Despite the promising overall survival outcomes using EPD, the current evidence is drawn from retrospective studies or meta-analyses, and there has not been randomized control trials evaluating EPD vs. EPP in a prospective manner. While there is still no consensus on which surgical procedure is better, NCCN guidelines state that although EPD and EPP are both considered curative-intent surgical options for PM, EPD may offer advantages over EPP, including lower rates of perioperative morbidity and mortality, as well as improved postoperative quality of life [16].

Surgery is part of a multimodal therapeutic approach and is recommended for early-stage PM, and the potentially beneficial effects of surgery have been investigated. The Mesothelioma and Radical Surgery (MARS) trial was a feasibility trial, where all patients initially underwent induction with platinum-based chemotherapy followed by clinical assessment, and after further consent, patients were randomly assigned (1:1) to EPP followed by postoperative hemithorax irradiation or to no EPP [20]. The main endpoints studied were the feasibility of randomly assigning 50 patients in 1 year, proportion randomized who received treatment, proportion eligible (registered) who proceeded to randomization, perioperative mortality, and quality of life. Treasure et al. demonstrated that the adjusted hazard ratio for overall survival between the EPP and non-EPP groups was 2.75 [95% CI 1.21–6.26], with no difference in the 12-month survival rate. They reported median survival was 14.4 months (5.3–18.7) for the EPP group and 19.5 months (13.4 to time not yet reached) for the no EPP group. [20]. As noted by a NCCN panel, the study results were controversial because overall survival was not the primary endpoint of the study. The MARS-2 trial compared treatment with chemotherapy and EPD versus chemotherapy alone. A total of 335 participants were randomly assigned to surgery and chemotherapy (n = 169) versus chemotherapy alone (n = 166). This randomized trial demonstrated that EPD with chemotherapy was associated with worse survival (median survival 19.3 months) as compared with chemotherapy alone (median survival 24.8 months). In addition, EPD with chemotherapy was associated a higher rate of adverse events among patients with resectable PM as compared with platinum and pemetrexed chemotherapy alone [21,22]. Ripley et al. have discussed the limitations of the MARS study, and recommended exercising caution when interpreting the results of the MARS 2 trial, as patients appropriate for surgery may be overlooked [15].

### 2.2. Hyperthermic Intrathoracic Chemotherapy

Hyperthermic intrathoracic chemotherapy, or HITHOC, is a widely used method of multimodality treatment that includes the intraoperative intrapleural injection of heated cytotoxic drugs, such as cisplatin, doxorubicin, and mitomycin C [23]. In patients with resectable PM, it is administered directly into the thoracic cavity after surgical resection of PM to target residual cancer cells while minimizing systemic toxicity. HITHOC is usually performed with a standard infusion time between 60 and 90 min at 41–43 °C after cytoreductive surgery is achieved. The rationale for intrathoracic chemotherapy is to increase the local cytotoxic effect on tumor cells and minimize systemic adverse effects. Hyperthermia not only improves the efficacy of chemotherapy by increasing drug absorption and drug action but also promotes protein denaturation in cancer cells, leaving nonmalignant tissue cells intact and unharmed [24,25]. The protein denaturation in cancer cells further enhances tumor cell apoptosis, contributing to the local cytotoxic effects.

Despite the wide usage of HITHOC and its well-established counterpart hyperthermic intraperitoneal chemotherapy (HIPEC) in the treatment of peritoneal carcinomatosis, there are no randomized controlled trials on the use of HITHOC when treating pleural malignancies, and mixed evidence exists on the benefits of HITHOC in the treatment of PM. In a Phase II prospective study by Tilleman et al., the median survival of the 92 patients treated with HITHOC was longer than that of the 29 patients treated without HITHOC (13.1 vs. 1.0 months, *p* = 0.01) [26]. While Tilleman et al., Sugarbaker et al., and Hod et al. reported improved survival with surgery and HITHOC as compared with surgery without HITHOC, Van Sandick et al. found markedly worse survival with HITHOC and either EPP or EPD without radiation therapy as compared with EPP and hemithoracic radiation therapy without HITHOC [24,26,27,28]. A meta-analysis by Zhao et al. demonstrated that the average median survival time was significantly longer in patients treated with HITHOC as compared with patients without HITHOC (Hedges’s g = 0.384 ± 0.105, 95% CI: 0.178~0.591, *p* < 0.001) [11]. Jarvinen et al. performed a meta-analysis which showed that even though there is evidence that there are benefits associated with HITHOC in patients with PM, higher-quality data are needed to make a definitive conclusion [10]. Currently, both the ESMO and NCCN do not include HITHOC within the recommended standard treatment for PM [7,8,16].

### 2.3. First-Line Systemic Therapy

Chemotherapy for PM: Patients who are not candidates for MCR will be managed non-surgically with systemic therapy. A combination of platinum and pemetrexed or raltitrexed is considered the standard first-line chemotherapy for PM. Vogelzang et al. demonstrated in their Phase III clinical trial that the addition of pemetrexed to cisplatin chemotherapy increased the median overall survival (12.1 vs. 9.3 months, HR 0.77, *p* = 0.02) [29]. Another Phase III clinical trial, by Van Meerbeeck et al., showed improved overall survival with raltitrexed and cisplatin as compared with cisplatin monotherapy [30]. Carboplatin–pemetrexed is a reasonable alternative to cisplatin–pemetrexed with comparable efficacy and is supported by the CHECKMATE-743 trial and the PM pemetrexed International Expanded Access Program [31].

Monoclonal Antibodies for PM: The Phase III Mesothelioma Avastin Cisplatin Pemetrexed Study (MAPS) demonstrated an improved median overall survival with the addition of the vascular endothelial growth factor (VEGF) monoclonal antibody bevacizumab to cisplatin–pemetrexed (18.8 versus 16.1 months, HR 0.77, *p* = 0.0167) [32,33]. Despite the observed benefit in overall survival, bevacizumab has not been FDA-approved for the treatment of PM. Studies looking at other ways to target VEGF have failed to show any improvement in overall survival. The CHECKMATE-743 trial showed superior efficacy for dual immunotherapy combining PD-L1 and cytotoxic T-lymphocyte antigen 4 (CTLA-4) inhibitors, recommending this as the new standard of care as compared with chemotherapy, especially in patients with non-epithelioid subtypes of PM [33,34,35]. These recent Phase II and III studies have laid the groundwork for shifting the focus from chemotherapy to immunotherapy as the preferred treatment for PM.

## 3. Immunotherapy for Treatment of PM

Immunotherapy, which harnesses the body’s own immune system to identify and attack cancer cells, has recently emerged as a promising new treatment modality for PM. Key immunotherapy strategies currently being explored in the field include immune checkpoint inhibitors (ICIs), dendritic cell (DC) vaccines, chimeric antigen receptor (CAR)-T cell therapy, and combination therapies.

### 3.1. Immune Checkpoint Inhibitors

Immune checkpoints are “brakes” that normally prevent the host immune system from attacking the body’s own tissues, thereby maintaining self-tolerance and preventing autoimmunity. However, cancer cells can exploit these checkpoints to avoid being attacked by the immune system, allowing them to grow and spread unchecked.

Immune checkpoint inhibitors, such as pembrolizumab and nivolumab, block the function of programmed death-1 (PD-1) receptor, a protein receptor on the surface of T cells that normally interacts with PD-L1 expressed on cancer cells to prevent T cells from attacking the body’s own tissues, which includes cancer cells. By blocking this interaction, these drugs allow T cells to attack and destroy cancer cells [32]. Other ICIs, like ipilimumab, are CTLA-4 blocking antibodies, which block another protein receptor on T cells that normally inhibits T-cell activation. By blocking CTLA-4, ipilimumab enhances T-cell activation and proliferation, promoting a stronger immune response against cancer cells [36].

A combination of checkpoint inhibitors, such as nivolumab and ipilimumab, can have a synergistic effect that further enhances the immune response. This combination approach has shown improved outcomes in patients with various cancers, including melanoma and non-small cell lung cancer [37]. In patients with PM, pembrolizumab (PD-1 inhibitor) elicited 10–29% response rates in Phase II trials, but single-agent pembrolizumab was not superior to chemotherapy in the PROMISE-Meso trial [38]. A combination of other PD-L1 inhibitors and CTLA-4 inhibitors, such as nivolumab and ipilimumab, had shown superior efficacy as compared with chemotherapy in the CHECKMATE-743 trial, especially in non-epithelioid subtypes of PM, leading to FDA approval of these drugs in 2020 [31,33,34,35]. Combining PD-L1 inhibitors with CTLA-4 inhibitors may yield similar response rates as PD-L1 monotherapy, however [39]. Further, despite the promising efficacy of ICIs, they may not only cause constitutional side effects, such as fatigue, rash, and diarrhea [34,39], but can also cause complications such as myocarditis, neuropathy, and severe pneumonitis due to increased immune activity [35].

### 3.2. Dendritic Cell Vaccines

Dendritic cell (DC) vaccines harness the ability of dendritic cells to stimulate a strong immune response against tumor cells by presenting antigens to T cells. To make DC vaccines, DCs are harvested from the patient and loaded with tumor antigens, which are derived from tumor lysates, specific tumor-associated proteins, or peptides, in a lab [40]. Once loaded, these DCs are matured and reinfused into the patient to present these antigens to the patient’s existing T cells, effectively priming the immune system to recognize and attack cancer cells [38]. Mature DCs are highly effective at activating naive T cells [41]. Upon reinfusion, these activated DCs migrate to the lymph nodes, where they present the tumor antigens to T cells. This process stimulates the proliferation of antigen-specific cytotoxic T lymphocytes, which can then seek out and destroy tumor cells [41].

In patients with PM, DC immunotherapy has shown anti-tumor activity in some clinical studies, indicating its potential as a treatment option [39,42,43,44]. In 2010, Hegmans et al. administered autologous monocyte-derived DCs loaded with autologous tumor cell lysates to 9 patients with PM twice intravenously and once intradermally [45]. Out of 9 patients, 3 patients exhibited a partial response within the first 8 weeks, and 2 of these patients had undergone chemotherapy shortly before the start of the DC treatment, which may have influenced the results [45]. In 2016, Cornelissan et al. used the same type of DCs but combined them with cyclophosphamide, a drug that inhibits regulatory T cells [46]. This study included 5 post-surgical and 5 non-surgical PM patients. A partial response was observed in 1 of the non-surgical patients. Overall, 7 out of 10 patients lived longer than 24 months, with a mean survival time of 37 months, indicating promising overall survival outcomes [46].

Due to the challenges of obtaining proper autologous tumor cell lysates, including the time-consuming process and patient reluctance for multiple pleural biopsies, Aerts et al. investigated an alternative antigen source in 2018. DCs were pulsed with a spectrum of tumor-associated antigens derived from allogeneic tumor lysates from human mesothelioma cell line cultures [47]. This approach was tested in 9 patients with PM, including 5 who had been pretreated with chemotherapy. A partial response was observed in 2 patients, one treatment-naive and one pretreated, lasting 15 and 21 months respectively. All other patients experienced disease control, with a median overall survival exceeding 22.8 months [47]. A European randomized Phase II/III trial (DENIM) is ongoing to compare DC immunotherapy with best supportive care as maintenance treatment post-chemotherapy [48].

### 3.3. Chimeric Antigen Receptor (CAR)-T Cell Therapy

CAR-T cell immunotherapy involves modifying a patient’s T cells to express chimeric antigen receptors (CARs) that specifically target antigens on cancer cells. Once infused back into the patient, these engineered T cells recognize and bind to the cancer cells, leading to their destruction. CAR-T cell therapy has shown promise in treating various cancers and is currently being explored for the treatment of PM, with three Phase I clinical trials completed and ongoing trials assessing its efficacy in PM [42,49].

A trial conducted by Adusumilli et al. included 25 mesothelioma patients with advanced PM. The CAR-T cells used for this study were modified with the cell-surface antigen mesothelin, which is highly expressed in mesothelioma and other cancers, making it an attractive target for CAR-T cell therapy [50,51,52,53,54]. Intrapleural administration of CAR-T cells was combined with pembrolizumab for 18 of those patients, because animal models had shown that PD-1 inhibitors enhance CAR-T cell function [50]. The median overall survival in these patients was 23.9 months, with a 1-year overall survival of 83% [50].

The tumor microenvironment (TME) in PM can pose significant challenges for immunotherapy, such as immunosuppression and limited T-cell infiltration [53]. Strategies to overcome these challenges include local delivery of CAR-T cells, improved CAR designs, and combination therapies with PD-L1 checkpoint inhibitors and oncolytic viruses [53,54,55,56].

### 3.4. Other Immunotherapeutic Strategies

Less common immunotherapies, including immunotoxin therapy, anticancer vaccines, oncolytic viral therapy, and adoptive cell therapy, are also being assessed in clinical trials to examine their potential benefits in treating PM [57,58,59].

## 4. Immunotherapy as Adjunct to MCR with HITHOC

ICIs as an adjunct to surgical management are revolutionizing the treatment landscape for patients with various cancers, including PM, by prolonging survival. While Phase III data on ICIs are available for unresectable PM, there is currently also at least one published paper with Phase II data on the combination of immunotherapy and surgery with HITHOC [29,30,31,32,33,34,35,36,37,38,58]. Another article describes a protocol where immunotherapy is used after surgery for PM, and there are several ongoing Phase II trials investigating immunotherapy as an adjunct to MCR with HITHOC [59].

The Window of Opportunity trial by Lee et al. (NCT02592551), conducted at Baylor College of Medicine, was a Phase II, randomized study investigating neoadjuvant immunotherapy in resectable PM [58]. This trial compared durvalumab (anti-PD-L1) monotherapy to durvalumab plus tremelimumab (CTLA-4 inhibitor) combination therapy, followed by MCR that included HITHOC as part of the standard treatment. Out of 24 enrolled patients, 9 patients were randomized to monotherapy with durvalumab, 11 to combination therapy with durvalumab and tremelimumab, and 4 to control with no immunotherapy prior to surgery. At 34.1 months follow-up, combination therapy patients showed longer median overall survival as compared with monotherapy (not reached vs. 14.0 months) [58].

Tumor pathologic response occurred in 35.3% of patients receiving immunotherapy and surgery. By measuring CD57+ T cells in the tumor immune microenvironment after treatment, the study found that combination therapy mobilized populations of both CD57-expressing CD8 and CD4 effector memory T cells from the bone marrow to the circulation, which was particularly effective in remodeling the immune contexture. These systemic immune responses correlated with favorable clinical outcomes. However, grade ≥3 immunotoxicity was observed in 8% of patients who received monotherapy and 27% of patients who received combination therapy, indicating a need for careful monitoring for combination immunotherapy. Overall, this trial demonstrated the feasibility and potential efficacy of neoadjuvant immunotherapy in patients with resectable PM, suggesting it may improve outcomes as compared with surgery alone. These findings contribute significantly to the evolving landscape of PM treatment, highlighting the potential of immunotherapy as a valuable addition to the multimodal approach in managing this challenging disease [58].

The NICITA study (NCT04177953) is an ongoing Phase II, randomized, open-label, multicenter trial investigating the efficacy of nivolumab combined with chemotherapy in patients with early-stage PM who have already undergone extended pleurectomy and decortication, with or without HITHOC. While this study does not specifically evaluate the impact of combining HITHOC with immunotherapy after surgery, it may provide insights into potential differences in outcomes between patients who received chemotherapy with HITHOC versus those who did not [59].

Several ongoing Phase II clinical trials are investigating the use of combinatorial immunotherapy administered either before or after surgery. However, these studies do not specify whether HITHOC is included as part of the surgical approach, so the inclusion of HITHOC in these trials remains unclear. Since these trials started within the last 3 years, results are expected to be published in the next 5 years. Table 1 provides details on the clinical trial names and the specific monoclonal antibody regimens being tested. All of the clinical trials detailed use ICIs as part of the immunotherapy regimen, and one of these Phase II trials (NCT05647265) is investigating the efficacy of combined ipilimumab (anti CTLA-4) and nivolumab (anti PD-1) as a neoadjuvant therapy.

### Hyperthermia and Immunotherapy

Individually, hyperthermia and immunotherapy have each proven their efficacy in improving outcomes in patients with PM. The combination of hyperthermia and immunotherapy, such as HITHOC with ICIs, leverages the synergistic effects of both treatments to enhance anti-tumor efficacy. Hyperthermia can alter the tumor microenvironment by increasing the expression of adhesion molecules, such as ICAM-1, on high endothelial venules, facilitating lymphocyte–endothelial adhesion and leukocyte trafficking [60]. By increasing the permeability of the tumor vasculature by reducing the interstitial fluid pressure and stromal stiffness of immune cells, hyperthermia may improve the penetration of therapeutic agents into tumors, which, in combination with the activation of T cells by immunotherapy, can result in a more robust anti-tumor immune response [61]. In addition, by increasing the expression of immune checkpoint or co-stimulatory molecules like PD-L1 on tumor cells, hyperthermia has the potential to make tumors more responsive to ICIs [62]. This transformation has been proposed to potentially convert “cold tumors”, which are less responsive to immunotherapy, into “hot tumors” that are more susceptible to immune attack [62,63].

Conversely, hyperthermia-induced increased expression of intracellular heat shock proteins (HSPs) has also been shown to promote tumor growth and immune evasion. HSPs can upregulate various cytokines (e.g., IL-10, IL-6, TGF-ß), leading to pro-tumoral effects such as angiogenesis, which may render immunotherapies ineffective [64]. Despite existing studies that favor the combination of immunotherapy and HITHOC to treat PM, there is also emphasis on the need for further research on immunotherapy-related toxicities that can affect multiple organ systems [44,65]. As hyperthermia can both enhance and detract from the efficacy of immunotherapy, precise control over the application of hyperthermia via temperature and time is essential to maximize its therapeutic benefits while minimizing potential adverse effects when treating patients with PM.

Overall, the synergistic combination of HITHOC and immunotherapy exploits the complementary mechanisms of hyperthermia-induced immune activation and the targeted action of immunotherapy, offering a promising approach for enhancing cancer treatment outcomes. This approach should be further evaluated in Phase II and III clinical trials before drawing conclusions.

## 5. Conclusions

In conclusion, pleural mesothelioma remains a challenge due to its aggressive nature, long latency period, and resistance to conventional treatments. Despite advances in treatment modalities, such as surgery, chemotherapy, radiotherapy, and immunotherapy, improvement in outcomes has been limited. The combination of these approaches, particularly with emerging techniques like HITHOC and ICIs, offers a promising avenue for improving patient outcomes. In this review, we presented the potential mechanisms of action for HITHOC and immunotherapies, as well as the current ongoing Phase II clinical trials for multimodal treatment of resectable PM.

Ongoing and future clinical trials are crucial in refining the optimal protocols and assessing the true efficacy of these multimodal treatments. As immunotherapy becomes more integrated into standard treatment regimens, ongoing research will need to focus on long-term safety and monitoring. Managing immune-related adverse events and understanding their long-term impacts will be crucial for ensuring patient safety and treatment tolerability. Well-designed studies are required for investigating the efficacy of a multimodal strategy including neoadjuvant treatment, surgical treatment, HITHOC, and adjuvant therapy. These trials may assist us in optimizing patient selection and significantly improving outcomes in patients with mesothelioma.

## Figures and Tables

**Table 1:** List of ongoing Phase II clinical trials combining surgery with immunotherapy.

Trial ID	Study Name	Immunotherapy Tested	Study Start	Type of Study
NCT06155279	Induction Chemo + Immunotherapy in Resectable Epithelioid and Biphasic Pleural Mesothelioma (CHIMERA Study)	**Pembrolizumab**	2024	Phase II prospective, open-label, multi-site
NCT05647265	Neoadjuvant Immunotherapy in Sarcomatoid Mesothelioma	**Ipilimumab and Nivolumab**	2023	Phase II
NCT05380713	Surgery for Mesothelioma After Radiation Therapy Using Exquisite Systemic Therapy (SMARTEST)	**Tremelimumab and Durvalumab**	2022	Phase II non-blinded, randomized
NCT04177953	NICITA study protocol	**Nivolumab**	2019	Randomized phase III
NCT02592551	MEDI4736 Or MEDI4736 + Tremelimumab In Surgically Resectable MPM	**Durvalumab vs Durvalumab + Tremelimumab**	2016	Phase II, randomized

## Data Availability

Not applicable.
